# Thumb Hypoplasia

**Published:** 2015-12-21

**Authors:** Lohrasb Sayadi, Mustafa Chopan, Donald Laub

**Affiliations:** University of Vermont College of Medicine, Burlington, Vermont

**Keywords:** thumb hypoplasia, pollicization, congenital hand radial longitudinal hypoplasia, thumb reconstruction

## DESCRIPTION

**Figure 1:**
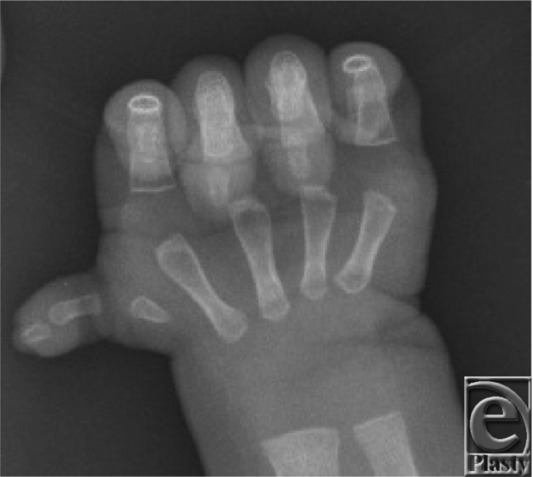
Radiograph of N.D. preoperatively, demonstrating thumb hypoplasia and an unstable carpometacarpal joint.

N.D. presented at 8 weeks of age because of concern over her small right thumb. She was unable to actively flex or oppose this thumb, and the first carpometacarpal joint was unstable. Radiographs showed a hypoplastic digit with undeveloped carpometacarpal joint (Fig. 1). The remainder of her examination showed normal findings.

## QUESTIONS

1. **When does a child start to develop function and opposition with the thumb?**
2. **What anomalies may be associated with thumb hypoplasia?**
3. **How are hypoplastic thumbs classified?**
4. **How and when are hypoplastic thumbs treated?**

## DISCUSSION

A child learns about the location and function of his or her thumb in the first few months of life and proceeds to fully utilize it by 9 months. At this point, the malformation of the thumb may hinder the child's quality of life. Loss of function, length, or stability of the thumb reduces precision, grip, span, and power all of which are important for carrying out daily activities.

Classic thumb hypoplasia is part of radial longitudinal deficiency and may be associated with amniotic band syndrome; Apert and Rubinstein-Taybi syndromes; Holt-Oram syndrome, thrombocytopenia-absent radius (TAR) syndrome; vertebral, anal, cardiac tracheoesophageal, renal, limb (VACTERL) syndrome; or Fanconi anemia.^1^^,^^2^ In addition, the psychological impact of having a hypoplastic thumb can become a serious stressor that can often be averted with surgical intervention.

The Blauth system has been used to classify thumb hypoplasia (Table 1). Patient N.B. presented with Blauth type IIIB hypoplastic right thumb. Characteristics of Blauth type III include partial aplasia of the first metacarpal that is typically more severe at the proximal end.^3^ The absence of motor units causes the thumb to lie against the second metacarpal. In type IIIB hypoplastic thumb, the proximal end of the first metacarpal is absent with more severe tendon and muscle deficiencies.^3^

**Figure 2:**
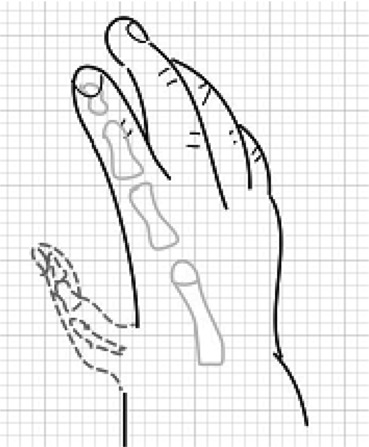
Schematic of the hand with hypoplastic thumb to be amputated (dashed lines).

**Figure 3:**
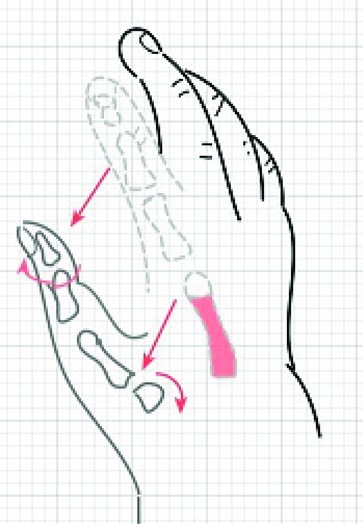
Schematic of hand demonstrating elements of pollicization: Shaft of index metacarpal excised (red), the index digit is moved proximally and radial (straight red arrows), as well as pronated (large curved red arrow), and the metacarpal head is flexed (small curved red arrow).

**Figure 4:**
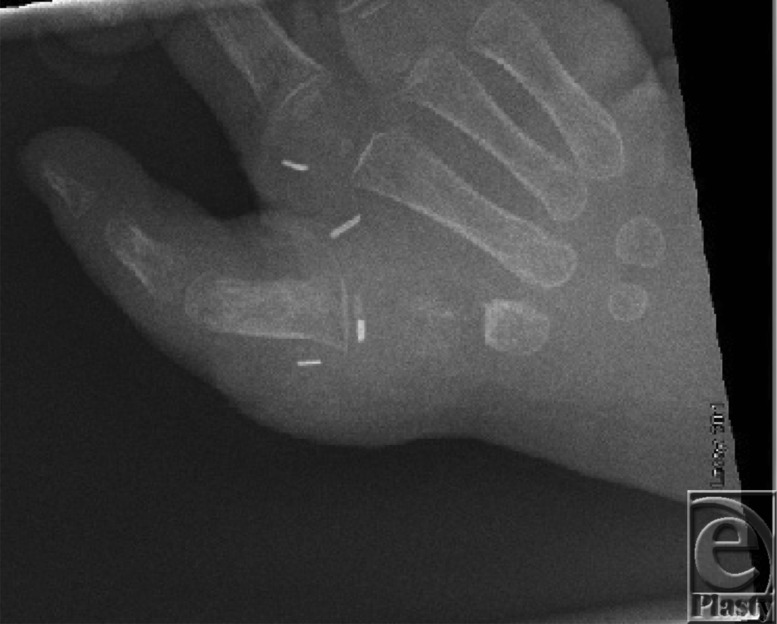
Radiograph of N.D. postoperatively, demonstrating the pollicized index finger.

Timing of pollicization is still somewhat controversial, but the current trend is to perform it in the first year of life.^4^ Some researchers argue that pollicization at a younger age takes advantage of brain plasticity and ease of incorporation of the thumb into daily activities.^5^ However, later presentation is not a contraindication; there is no evidence that functional results are dependent on the patient's age at operation.^6^ Our patient N.D. was 15 months of age at the time of pollicization. A team of an orthopedic surgeon and a plastic surgeon operated. The hypoplastic thumb was excised (Fig. 2). The shaft of index metacarpal was excised, preserving the metacarpal head (Fig. 3). The index digit was moved proximally and radially, as well as pronated, and the metacarpal head was flexed Figure 4. To stabilize in the correct position, a Kischner wire and interrupted sutures were placed. Three Y-V flaps were designed to deepen the web space.

Occupational therapy following pollicization focuses on thumb usage, with the initial goal of large object manipulation and ultimately to fine pinch.^5^ An active range-of-motion program is started without any restraints, and the patient it followed closely for the first year.^2^ The development of postoperative adhesions is rare in children and is usually addressed with aggressive hand therapy.^2^ Neuronal plasticity, the development of new neuronal connections and sprouting of adjacent synapses, and motor relearning play a pivotal role in rehabilitation.^5^ In addition, studies have found that 15 years after surgery, patients with early thumb pollicization achieve near-normal control of fingertip forces that plays a key function in the child's dexterity and in-hand objection manipulation.^7^ Patients N.D.'s quality of life significantly improved after surgical intervention. Pollicization coupled with aggressive therapy has given N.D. the ability to have strength and opposition in her newly formed thumb.

## Figures and Tables

**Table 1:** Modified Blauth classification of thumb hypoplasia*

Type	Description	Treatment
Type I	Minor hypoplasia	No treatment required
	All musculoskeletal and neurovascular components of the digit are present but small in size	
Type II	All of the osseous structures are present (may be small)	Stabilization of the MCP joint
	MCP joint ulnar collateral ligament instability	Release of the first web space
	Thenar hypoplasia	Opponensplasty
Type IIIA	Musculoskeletal and osseous deficiencies	
	CMC joint intact	
	Absence of active motion at the MCP or IP join	
Type IIIB	Musculoskeletal and osseous deficiencies	
	Basal metacarpal aplasia with the deficient CMC joint	
	Absence of active motion at the MCP or IP joint	Thumb amputation and pollicization
Type VI	Floating thumb	
	Attachment of the hand by the skin and digital neurovascular structures	
Type V	Complete absence of the thumb	

* MCP indicates metacarpophalangeal; CMC, carpometacarpal; IP, interphalangeal.
